# Effects of Gaze Stabilization Exercises on Gait, Plantar Pressure, and Balance Function in Post-Stroke Patients: A Randomized Controlled Trial

**DOI:** 10.3390/brainsci12121694

**Published:** 2022-12-09

**Authors:** Ruoxin Zhao, Jun Lu, Yue Xiao, Xinrong Liu, Yu Wang, Guangxu Xu

**Affiliations:** 1 Department of Rehabilitation Medicine, Nanjing Medical University, Hanzhong Road 140, Nanjing 210029, China; 2 Rehabilitation Medicine Center, The First Affiliated Hospital of Nanjing Medical University, Guangzhou Road 300, Nanjing 210029, China

**Keywords:** vestibular rehabilitation, stroke, balance, gaze stabilization exercises, gait, plantar pressure

## Abstract

This study aims to explore the effects of gaze stabilization exercises (GSEs) on gait, plantar pressure, and balance function in post-stroke patients (≤6 months). Forty post-stroke patients were randomly divided into an experimental group (*n* = 20) and a control group (*n* = 20). The experimental group performed GSEs combined with physical therapy, while the control group only performed physical therapy, once a day, 5 days a week, for 4 weeks. The Berg Balance Scale (BBS) was used to test the balance function and the risk of falling, which was the primary outcome. The Timed Up and Go test (TUGT) evaluated the walking ability and the fall risk. The envelope ellipse area and the plantar pressure proportion of the affected side were used to measure the patient’s supporting capacity and stability in static standing. The anterior–posterior center of pressure displacement velocity was used to test the weight-shifting capacity. Compared to the control group, the swing phase of the affected side, swing phase’s absolute symmetric index, envelope ellipse area when eyes closed, and TUGT of the experimental group had significantly decreased after GSEs (*p* < 0.05); the BBS scores, TUGT, the anterior–posterior COP displacement velocity, and the plantar pressure proportion of the affected side had significantly increased after 4 weeks of training (*p* < 0.05). In conclusion, GSEs combined with physical therapy can improve the gait and balance function of people following stroke. Furthermore, it can enhance the weight-shifting and one-leg standing capacity of the affected side, thus reducing the risk of falling.

## 1. Introduction

Over the past 30 years, the burden caused by stroke has greatly increased, which has been one of the most common causes of death worldwide [1]. After a stroke, reduced control of both body and gaze movements can lead to decreased performance during walking and turning [2]. Gait asymmetry was observed in over 30% of individuals with stroke [3]. Compared with the unaffected lower extremity, the affected side of people following stroke exhibits prolonged swing phase duration and shortened single-limb support time. When walking straight, lower-limb movements ensuing forward body progression induce a rotational motion of the pelvis in the horizontal plane [4]. Unfortunately, people following stroke display abnormally large head and gaze yaw motions with respect to space, indicating a defect in stabilization mechanisms. Such a phenomenon could be observed in all stroke individuals but was more pronounced in the low-functioning ones walking at slower speeds [5].

A specialized form of vestibular rehabilitation, gaze stabilization exercises (GSEs) include substitution and adaptation exercises based on the vestibulo-ocular reflex (VOR). Substitution exercises are developed to promote alternative strategies (saccadic eye movements and smooth tracking exercises) to substitute for missing vestibular function. Adaptation exercises refer to long-term changes in the neuronal response to head movements with the goal of reducing symptoms and normalizing gaze and postural stability [6]. Evidence has shown that GSEs can improve the balance function, confidence, and cognition in older adults with mild cognitive dysfunction [7]. In addition, GSEs can also improve postural stability in healthy adults [8]. The underlying principle is that GSEs can greatly benefit active VOR and dynamic visual acuity. VOR originates in the vestibular nuclei and passes through the posterolateral thalamus to the temporal–insular cortex. Impairment in the structure and function of any of these structures might decrease gaze stabilization, and vestibular compensation then occurs [9]. Vestibular compensation occurs through three mechanisms: adaptation, substitution, and habituation, which include a rapid vestibulocentric static process and a longer-term, dynamic, distributed learning process. GSEs correlate highly with the first two mechanisms (adaptation and substitution) [10]. Brain remodeling can be induced at any age, and neuronal reorganization depends on experience. Mechanisms of experience-dependent plasticity contribute to brain reorganization after stroke and the efficacy of rehabilitation exercises [11]. The plastic remodeling of synaptic connections follows the Hebbian principle of neural network plasticity, which shows that when the brain is engaged in behavior, simultaneously activated inputs increase in tandem and improve their synergy [12].

The VOR is a critical step of gaze stabilization. Gaze stabilization and orientation are important in establishing a frame of reference during steering [13]. When the head moves, the VOR stabilizes the gaze (eye position in space), producing eye movements of equal speed and in a direction opposite to the movement of the head to ensure satisfactory visual acuity [14]. The VOR controls eye movements to preserve clear visual acuity by altering the retinal slip related to head influence amid strolling [15]. The influence of the head increases incrementally with the increase in walking speed; quick strolling requires coordination of the head as well as the VOR [16]. VOR gain has a certain effect on the balance and posture of people following stroke.

GSEs may also activate the excitability of the vestibulospinal tract (VST) and enhance the sensory input to the medial VST (MVST) to promote the head–eye coordination of people following stroke [17]. The VST is an extrapyramidal motor pathway to the spinal cord and controls balance [18]. The vestibular nuclei influence postural control via the lateral/medial vestibulospinal tracts and the reticulospinal tract [19]. The MVST terminates primarily in the upper cervical regions of the spinal cord and plays a dynamic role in voluntary gaze stabilization and eye/head tracking; it is important for human postural control. The excitability of MVST may also be affected by different postural control [20]. It processes visual information and decides which movements are required to control the balance of an upright posture. The anterolateral VST tract and MVST tract were found to be significantly degraded in the elderly [21]. Miller et al. considered that damage to the VST may be the cause of impaired postural control seen in stroke [22].

Nowadays, GSEs are widely used in vestibular dysfunction and many other neurological disorders, such as dizziness, multiple sclerosis, and Parkinson’s disease [23,24,25]. Some studies also investigated the influence of GSEs on postural stability and fall risk of people following stroke [26,27]. However, the effectiveness of GSEs in the gait and plantar pressure of people following stroke is not clear. Therefore, this single-blinded randomized control study aimed to investigate the effects of GSEs on balance function, gait, and plantar pressure in people following stroke.

## 2. Materials and Methods

### 2.1. Participants

The sample size was calculated using the G* power software (v3.1.9.2, University Dusseldorf, Düsseldorf, Germany), based on a comparison of outcome measures between the GSEs and control groups, represented by a minimum increase of four points in the Berg Balance Scale [28]. According to a prior two-way analysis of variance (ANOVA) F-test, with a power of 0.80, an alpha level of 0.05, and a fall rate of 20%, this study required an estimated 40 participants.

Individuals were included if they (1) had a stroke within the past 6 months, diagnosed by computed tomography (CT) or magnetic resonance imaging (MRI; ischemic infarction or intracerebral hemorrhage) [29]; (2) were between 18 and 75 years old; (3) had a one-sided hemiparesis but could walk independently (with or without assistance ≥ 3 m); (4) could understand and follow instructions (MMSE > 23); (5) signed an informed consent form.

Individuals were excluded if they had (1) significant musculoskeletal or other neurological disorders diagnosed by CT or MRI; (2) balance dysfunction before the stroke (BBS < 40); (3) oculomotor nerve defects or presence of eye movement disorders, spontaneous or nonspontaneous nystagmus through clinical examinations [30]; (4) severe unilateral spatial neglect assessed by the Line Bisection Test [31] or severe aphasia assessed by The Boston Diagnostic Aphasia Examination [32].

### 2.2. Study Design

This study was a prospective, assessor-blinded clinical randomized controlled trial registered on ClinicalTrials.gov (accessed on 12 November 2022) (ChiCTR2100052242). All the participants were recruited from the Department of Rehabilitation Medicine of The First Affiliated Hospital of Nanjing Medical University and Nanjing Qixia District Hospital between June 2021 and January 2022. The ethics committee of The First Affiliated Hospital of Nanjing Medical University approved the experimental procedures (2021-SR-213). This study was a single-blind randomized controlled trial and the assessors were also blinded. The same physiotherapist conducted all the training. The assessor did not know how the participants were grouped.

**Figure 1:**
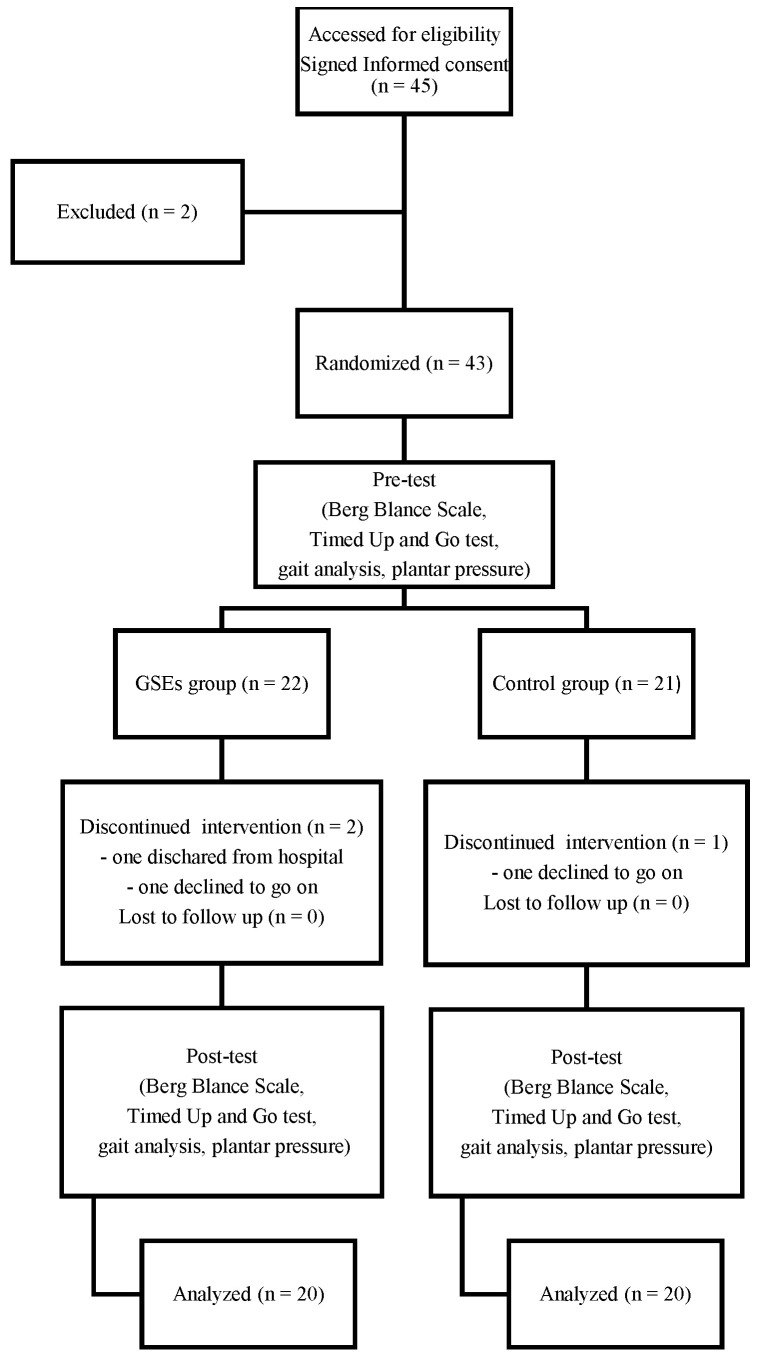
Flow diagram for subject assignment in the study.

The study flow chart is shown in Figure 1. A total of forty-five participants were enrolled in this trial. Two participants were excluded before the randomization. Forty-three participants were randomized in a 1:1 ratio to either a customized 4-week GSE group (GG) or a control group (CG), utilizing freely available software (Research Randomizer 4.0). Three participants dropped the trial because they were discharged or refused to continue (GG = 2, CG = 1). The compliance in GG was approximately 90.9% and in CG was 95.2%. Thus, 40 participants were finally included in the analysis (GG = 20, CG = 20).

Participants were first diagnosed with stroke by an experienced neurological physician through CT or MRI. After receiving an explanation of the experimental protocol, the participants were required to sign a written informed consent form to participate in this study.

### 2.3. Gaze Stabilization Exercises

**Figure 2:**
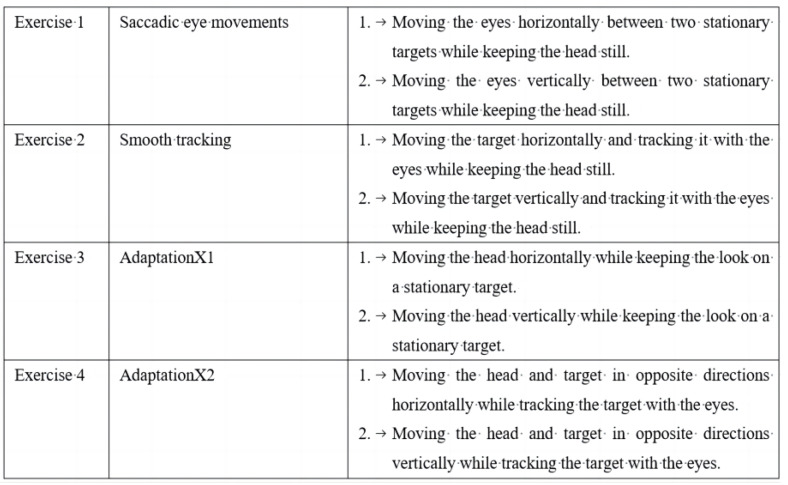
The detailed procedures of gaze stabilization exercises.

The GG performed GSEs for 4 weeks (5 times a week) and each exercise lasted for 5 min. After the first 2 exercises, there was a 10 min rest. The entire session lasted 30 min (Figure 2).

The training was conducted in inpatient department settings with white walls as the background to reduce visual interference with the attention of the participants. The participants remained standing during the training, except for the rest. An experienced nurse closely observed changes in the participant during the intervention and carefully inquired whether the participant had any discomfort or any problems.

### 2.4. Physical Therapy Treatments

Both the GG and CG were provided with physical therapy treatments for 30 min (including a 10 min rest), once a day, 5 days a week, for 4 weeks. The treatment plan was designed to improve postural stability and mobility through (1) standing balance training, (2) weight shifting training, (3) walking training, (4) muscle strength training, and (5) step-up-and-down training. Both groups received physical therapy treatments of the same difficulty.

### 2.5. Primary Outcome

The Berg Balance Scale (BBS): The BBS is a 14-item scale that quantitatively assesses balance and risk for falls through direct observation of their performance. It is highly sensitive to changes in people following stroke [33]. Patients who score 46 or less have a high probability of falls [34].

### 2.6. Secondary Outcomes

Timed Up and Go test (TUGT): The TUGT measures the time taken for a patient to get out of a chair, walk straight for 3 m, turn, walk back to the chair, and sit down [35] TUGT is highly associated with lower-limb impairments and locomotor capacities of people following stroke [36].

The other secondary outcomes were assessed by the ODONATE gait analysis system (ODONATE, Maver, Shanghai, China), which is highly reliable and valid in gait analysis, according to a previous study [37]. A three-dimensional binocular vision sensor measurement camera was used to capture the movement of both feet. The three-dimensional model of human motion was reconstructed and calculated by deep neural network technology.

Gait analysis: The gait analysis results include the stance phase (ST) of both sides, swing phase (SW) of both sides, and absolute symmetry index (ASI):

$$\mathrm{ASI}=\frac{\mathrm{Vaffected}-\mathrm{Vunaffected}}{0.5\;\left(\mathrm{Vaffected}\;+\mathrm{Vunaffected}\right)}$$

The value of ASI represents the degree of asymmetry. An ASI value equal to 1 reflects full symmetry. ASI was calculated for ST and SW (ST-ASI and SW-ASI).

**Figure 3:**
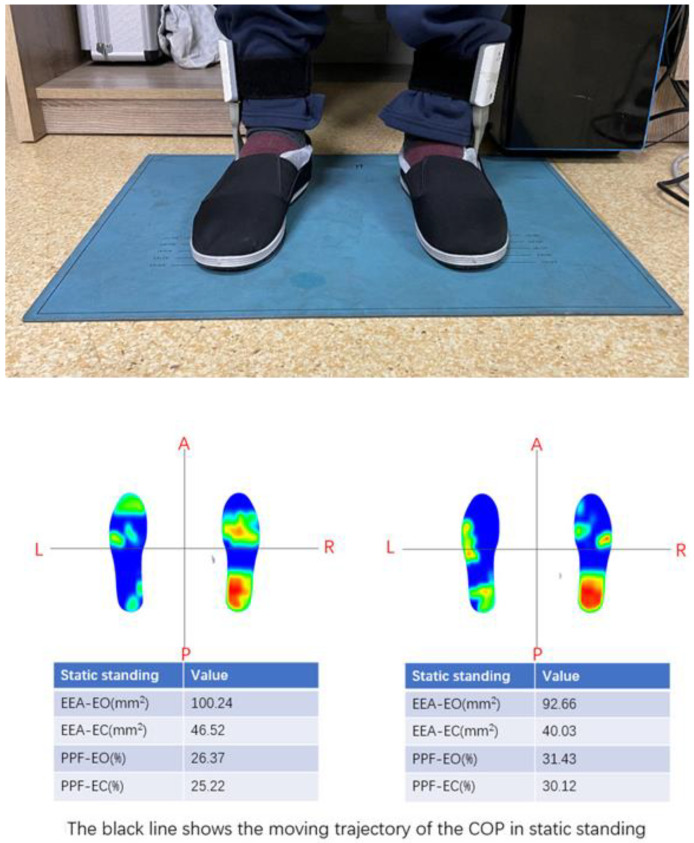
A typical example of static plantar pressure measured by the ODONATE system.

Plantar pressure: During the evaluation process, the participants needed to wear a special pressure insole and step on the pressure mat. The plantar pressure was determined using a pair of sensors attached to the pressure insole. The proportion of the plantar pressure of the affected foot (PPF-EO: eyes open; PPF-EC: eyes closed) and envelope ellipse area (EEA-EO: eyes open; EEA-EC: eyes closed) of each participant was obtained by standing neutrally on the pressure mat for 20 s with eyes open and then 20 s with eyes closed. They were used to measure the supporting capacity and stability of the participants in static standing (Figure 3).

**Figure 4:**
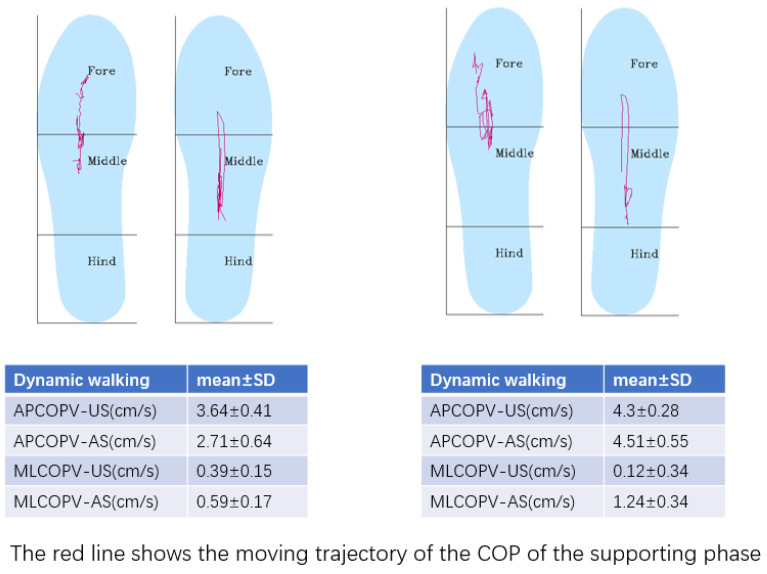
A typical example of center of pressure displacement measured by the ODONATE system.

During dynamic walking, the anterior–posterior velocity of each participant’s center of pressure displacement (APCOPV-US: the unaffected side; APCOPV-AS: the affected side) reflects the ability of dynamic balance and mobility of the participants and was determined by the binocular vision sensor measurement camera and the pressure sensors (Figure 4).

### 2.7. Data and Statistical Analysis

The IBM SPSS Statistics software (v26, IBM Corp., Armonk, NY, USA) was used for statistical analysis. Normality was checked using the Shapiro–Wilk test. Baseline characteristics were compared among groups using Student’s *t*-test for quantitative variables and the chi-square test for qualitative variables. Within-group differences in pre-and post-training that were normally distributed were analyzed using paired *t*-tests. One-way ANOVA was used to compare between-group differences in pre-and post-training. Effect sizes were determined by converting partial eta-squared (η^2^_p_), which was the most frequently used [38]. For those that were not normally distributed, the Mann–Whitney U tests were used to compare the pre-and post-training between-group differences. Additionally, the alpha level of statistical significance was set at 0.05 for all the tests.

## 3. Results

### 3.1. Baseline Demographic and Clinical Characteristics

The characteristics of the participants are presented in Table 1. There were no significant differences between the two groups (GG and CG) (*p* > 0.05). During the study period, no adverse events were observed.

### 3.2. Primary Outcome

#### The Berg Balance Scale

BBS (CG = 48.35 ± 6.65, GG = 49.05 ± 4.70, *p* < 0.001) was significantly different between CG and GG and improved compared to pre-training (Table 2).

### 3.3. Secondary Outcomes

#### 3.3.1. Timed Up and Go Test

TUGT (CG = 23.03 ± 8.79 s, GG = 22.83 ± 11.14 s, *p* < 0.001) was significantly different between CG and GG and improved compared to pretraining (Table 2).

#### 3.3.2. The Temporal and Spatial Characteristics of Gait

Gait characteristics are mentioned in Table 2. SW-AS (CG = 0.53 ± 0.15 s, GG = 0.45 ± 0.14 s, *p* = 0.041) and SW-ASI (CG = 32.65 ± 25.91, GG = 30.57 ± 33.08, *p* = 0.025) of the GSEs group were significantly decreased between groups and compared to the pretraining values.

#### 3.3.3. Plantar Pressure

At baseline, no statistically significant difference was observed between the two groups (*p* > 0.05). In some cases, differences between the two groups were not normally distributed, and nonparametric statistical tests were applied for these variables, denoted with a superscript ^m^ in the last column of Table 3. Specifically, significant differences were found for the APCOPV-US (CG = 11.13 ± 4.46 cm/s, GG = 12.03 ± 6.83 cm/s, *p* = 0.018), APCOPV-AS (CG = 10.80 ± 4.27 cm/s, GG = 11.82 ± 5.46 cm/s, *p* = 0.013), EEA-EC (CG = 146.28 ± 136.65 mm^2^, GG = 73.21 ± 72.40 mm^2^, *p* = 0.014), PPF-EO (CG = 48.71 ± 18.48%, GG = 60.28 ± 17.86%, *p* = 0.037), and PPF-EC (CG = 48.19 ± 18.75%, GG = 55.04 ± 13.77%, *p* = 0.043) between groups (*p* < 0.05). In the GG, APCOPV-AS, PPF-EO, and PPF-EC varied significantly post-training than in the pretraining stage, but this was not so in the CG group (*p* < 0.05).

## 4. Discussion

This study provides a unique insight into the influence of GSEs on the balance function, gait, and plantar pressure of people following stroke. The findings suggest that after four weeks of GSEs, the swing phase of the affected side, the ASI of the swing phase, and the envelope ellipse area of the GSEs group had significantly decreased when the eyes closed. Furthermore, compared to the control group, the BBS scores, the anterior–posterior COP displacement velocity of the affected side, and the plantar pressure proportion of the affected side of the GSEs group had significantly increased after GSEs.

### 4.1. Balance and Fall Risk

Stroke-related motor, sensory, and visual deficits are associated with mobility impairments [39]. Such mobility impairments include falls, which are common and serious medical complications after a stroke [40]. Falls may increase the fear of falling and may subsequently lead to physical activity reduced, which can result in physical deconditioning and may further increase the risk of falls in the long term [41]. Therefore, it is highly important to take measures to prevent falls. Park et al. showed that vestibular rehabilitation based on eye movement could improve static and dynamic balance functions and reduce the risk of falls in older adults with a history of falls [42]. In this study, we employed BBS and TUGT to investigate the effectiveness of GSEs on the balance function and the risk of falls of people following stroke. BBS is an effective and appropriate assessment of balance in patients with stroke [43]. A review suggests that if an individual has a BBS score over 20 and experiences a change of between 3 and 7, one can be 95% confident that there has been a real improvement in balance [44]. In addition, TUGT is highly correlated with gait performance and walking endurance in subjects with stroke [36]. According to our results, the sit-to-stand transfer ability and walking performance after GSEs were greatly improved, reflected in the significant reduction in TUGT. Moreover, BBS scores in the GSEs group increased by an average of nearly six points, two points higher than that of the control group, which indicates that GSEs can significantly improve patients’ balance function and reduce the risk of falls. The improvement in BBS scores was reflected mainly in the ability to stand statically with eyes closed and one-leg standing time, and this is consistent with the remainder of our results.

### 4.2. Gait Performance

In post-stroke gait, the asymmetric pattern with higher kinetic involvement of the unaffected side may be a specific adaption to the initial and ongoing paresis of the affected side. This adaptation may involve both automatic and intentional cognitive processes, to provide support, balance, and progression purposes [45]. The asymmetry of movement between the affected and unaffected sides in people following stroke is an important factor affecting walking ability and postural stability; it is also one of the main causes of falls [46]. Compared with the general population, people following stroke have significantly reduced step frequency, step speed, step length of the affected side, shortened swing phase on the affected side, and decreased ability of center of gravity transfer. They also have prolonged gait cycles and support phases of the affected side [47]. In our study, the proportion of the swing phase of the affected side decreased significantly, which can be explained by the improved supporting capacity of the affected side after GSEs. During GSEs, the proprioception of the affected lower limb is constantly stimulated and the COP is often tilted to the affected side. Consequently, the patients can greatly benefit from repeated exercises that strengthen the COP transfer.

### 4.3. Plantar Pressure and Center of Pressure

Owing to their reduced movement, people following stroke show asymmetrical plantar pressure distribution and excessive postural sway while standing. The sway of their bodies increased approximately two-fold and their ability to stabilize decreased compared to the static standing posture of normal subjects. This increased swaying of the body in static standing leads to asymmetrical loading of the weight on both lower extremities and decreases the ability to move the center of gravity toward the affected side, which leads to instability of gait, decreased gait speed, and increased risk of falls [48]. After GSEs, the body sway of the participants in static standing decreased, similar to that reported by Mitsutake [27].

Post-stroke patients also tend to have insufficient supporting ability and reduced ability to shift the center of gravity on the affected side. Kamono and Ogihara demonstrated that the support phase duration of the stroke-affected leg is much shorter than that of the contralateral leg at low walking velocities [49]. Consequently, the time to swing the unaffected leg forward is not enough, and the step length of the unaffected leg is substantially shorter compared to that of the affected leg in patients with low walking velocity. Therefore, to increase walking velocity, the step length of the unaffected leg should particularly be improved by extending the duration of the support phase of the affected leg to allow sufficient time for the contralateral leg to step forward. Under this condition, the affected leg must exert a larger upward impulse to cope with the downward impulse due to gravity during the swing phase of the unaffected leg. We observed that after GESs, APCOPV decreased significantly, showing the improved ability of anterior and posterior transference of the body weight. During walking, the trajectory of COP on the sagittal plane reflected the ability to control the movement of body weight and walking.

The process of GSE implementation is convenient and can be performed in many settings, such as a home-based environment or virtual reality, and requires less manpower and resources. Therefore, gaze stabilization exercises may be incorporated into any rehabilitation program with significant gains in improving balance and mobility.

### 4.4. Limitations

This study had a few limitations that need to be acknowledged. First, the sample size was relevantly small, so some data did not fit a normal distribution. Second, GSEs may have different influences on specific stroke lesions, such as the vestibulospinal tract and the parietoinsular vestibular cortex. The basal ganglia, the cerebellum, the hippocampus, and subdivisions of the thalamus are all suggested to be part of the visual–vestibular network [50]. Hence, it is essential to have a comprehensive clarity of the vestibular pathways involved to understand the consequences and develop therapeutic strategies to facilitate recovery. Consequently, imaging localization could be used in these areas in the future. Third, the VOR gain needs to be accurately measured with specialized instruments. VOR gain is mainly used to quantify the effect of GSEs in clinical studies. Future studies are thus still needed to prove the precise VOR gain after GSEs in people following stroke. Finally, the principle of rehabilitation therapy should vary from person to person, in a step-by-step manner. However, in our study, GSEs were implemented based on conventional physical therapy treatments, and the intervention programs were not personalized. It could be interesting for further studies to also investigate the effects of a new GSE paradigm with progressed procedures.

## 5. Conclusions

The present study demonstrated that GSEs combined with physical therapy treatments can improve the gait and balance function of people following stroke. Furthermore, it can improve the weight-shifting and one-leg standing capacity of the affected side, thus reducing the risk of falls.

## Figures and Tables

**Table 1:** Baseline demographic and clinical characteristics.

Baseline Characteristics	Control Group	GSEs Group	t/χ^2^	*p*-Value
Age (year)	54.45 ± 13.94	60.4 ± 12.32	1.430	0.161
Gender (*n*, male/female)	15/5	15/5	0.000	1.00
Height (m)	1.69 ± 0.07	1.68 ± 0.08	−0.381	0.705
Weight (kg)	71.11 ± 9.51	68.75 ± 11.47	−0.929	0.359
BMI (kg/m^2^)	25.20 ± 2.72	23.69 ± 2.95	1.682	0.101
Disease duration (month)	3.35 ± 1.87	3.95 ± 2.3	−0.904	0.372
ADL (score)	69.35 ± 11.26	67.65 ± 13.61	−0.937	0.355
FMA-LE (score)	23.26 ± 2.64	22.55 ± 3.02	−0.731	0.469
Affected side (*n*, left/right)	9/11	6/14	0.960	0.327
Stroke type (*n*, infarction/hemorrhage)	12/8	14/6	0.440	0.714
Lesion locations (*n*, basal ganglia region/cerebral hemisphere)	12/8	13/7	0.107	0.744

ADL: scale of activities of daily living; BMI: body mass index; FMA-LE: Fugl–Meyer motor function assessment—lower limbs.

**Table 2:** The temporal and spatial characteristics of pre-and post-training gait.

	Pretraining			Post-Training		
	Control Group (Mean ± SD)	GSEs Group (Mean ± SD)	*p*-Value	Control Group (Mean ± SD)	GSEs Group (Mean ± SD)	*p*-Value (Effect Size)
BBS (score)	44.25 ± 6.94	43.35 ± 4.89	0.135	48.35 ± 6.65 ^a^	49.05 ± 4.70 ^a^	**<0.001 (0.339)**
TUGT (s)	28.60 ± 11.80	28.99 ± 12.31	0.419	23.03 ± 8.79 ^a^	22.83 ± 11.14 ^a^	<0.001 ^m^ (0.255)
ST-US (s)	1.55 ± 0.65	1.67 ± 0.81	0.595	1.40 ± 0.54 ^a^	1.42 ± 0.65 ^a^	0.307 (0.027)
ST-AS (s)	1.50 ± 0.66	1.51 ± 0.69	0.959	1.30 ± 0.48 ^a^	1.31 ± 0.48 ^a^	0.970 (0.001)
SW-US (s)	0.37 ± 0.96	0.34 ± 0.55	0.129	0.41 ± 0.12 ^a^	0.36 ± 0.12	0.690 (0.004)
SW-AS (s)	0.50 ± 1.35	0.53 ± 2.77	0.785	0.53 ± 0.15	0.45 ± 0.14 ^a^	**0.041 (0.105)**
ST-ASI	9.42 ± 9.06	10.94 ± 8.42	0.586	11.56 ± 8.15	12.53 ± 9.75	0.862 (0.001)
SW-ASI	32.96 ± 23.94	39.98 ± 31.10	0.160	32.65 ± 25.91	30.57 ± 33.08 ^a^	**0.025 (0.126)**

SD: standard deviation; BBS: Berg Balance Scale; TUGT: Timed Up and Go test; ST-US: stance phase of the unaffected side; ST-AS: stance phase of the affected side; SW-US: swing phase of the unaffected side; SW-AS: swing phase of the affected side; ST-ASI: stance phase’s absolute symmetric index; SW-ASI: swing phase’s absolute symmetric index; ^a^: significant difference between PRE and POST; ^m^: Mann–Whitney U test was used; ^a^ significant difference between pre-and post-training; bolding: significant difference between two groups.

**Table 3:** The measurements of plantar pressure pre-and post-training.

	Pretraining			Post-Training		
	Control Group (Mean ± SD)	GSEs Group (Mean ± SD)	*p*-Value	Control Group (Mean ± SD)	GSEs Group (Mean ± SD)	*p*-Value (Effect Size)
APCOPV-US (cm/s)	10.76 ± 5.78	9.10 ± 5.91	0.374	11.13 ± 4.46 ^a^	12.03 ± 6.83 ^a^	**0.018 (0.139)**
APCOPV-AS (cm/s)	8.84 ± 4.95	8.30 ± 4.30	0.715	10.80 ± 4.27	11.82 ± 5.46 ^a^	**0.013 ^m^ (0.155)**
EEA-EO (mm^2^)	177.82 ± 256.47	205.02 ± 403.66	0.801	143.01 ± 111.49	153.15 ± 200.73	0.358 ^m^ (0.021)
EEA-EC (mm^2^)	234.57 ± 228.92	274.62 ± 184.22	0.546	146.28 ± 136.65 ^a^	73.21 ± 72.40 ^a^	**0.014 ^m^ (0.152)**
PPF-EO (%)	44.20 ± 18.12	47.22 ± 18.06	0.601	48.71 ± 18.48	60.28 ± 17.86 ^a^	**0.037 (0.110)**
PPF-EC (%)	43.87 ± 17.85	42.20 ± 12.99	0.737	48.19 ± 18.75	55.04 ± 13.77 ^a^	**0.043 (0.103)**

SD: standard deviation; APCOPV-US: anterior–posterior center of pressure displacement velocity of the unaffected side; APCOPV-AS: anterior–posterior center of pressure displacement velocity of the affected side; EEA-EO: envelope ellipse area (eyes open); EEA-EC: envelope ellipse area (eyes closed); PPF-EO: proportion of the plantar pressure of the affected foot (eyes open); PPF-EC: proportion of the plantar pressure of the affected foot (eyes closed); ^m^: Mann–Whitney U test was used; ^a^ significant difference between pre-and post-training; bolding: significant difference between two groups.

## Data Availability

Data can be made available by the corresponding author upon request.
